# Therapeutic Efficacy of Human Hepatocyte Transplantation in a SCID/uPA Mouse Model with Inducible Liver Disease

**DOI:** 10.1371/journal.pone.0009209

**Published:** 2010-02-18

**Authors:** Donna N. Douglas, Toshiyasu Kawahara, Banu Sis, David Bond, Karl P. Fischer, D. Lorne J. Tyrrell, Jamie T. Lewis, Norman M. Kneteman

**Affiliations:** 1 Department of Surgery, University of Alberta, Edmonton, Alberta, Canada; 2 Department of Medical Microbiology and Immunology, University of Alberta, Edmonton, Alberta, Canada; 3 Department of Laboratory Medicine and Pathology, University of Alberta Hospital, Edmonton, Alberta, Canada; 4 KMT Hepatech, Inc., Edmonton, Alberta, Canada; Yale University, United States of America

## Abstract

**Background:**

Severe Combined Immune Deficient (SCID)/Urokinase-type Plasminogen Activator (uPA) mice undergo liver failure and are useful hosts for the propagation of transplanted human hepatocytes (HH) which must compete with recipient-derived hepatocytes for replacement of the diseased liver parenchyma. While partial replacement by HH has proven useful for studies with Hepatitis C virus, complete replacement of SCID/uPA mouse liver by HH has never been achieved and limits the broader application of these mice for other areas of biomedical research. The herpes simplex virus type-1 thymidine kinase (HSV*tk*)/ganciclovir (GCV) system is a powerful tool for cell-specific ablation in transgenic animals. The aim of this study was to selectively eliminate murine-derived parenchymal liver cells from humanized SCID/uPA mouse liver in order to achieve mice with completely humanized liver parenchyma. Thus, we reproduced the HSV*tk* (vTK)/GCV system of hepatic failure in SCID/uPA mice.

**Methodology/Principal Findings:**

In vitro experiments demonstrated efficient killing of vTK expressing hepatoma cells after GCV treatment. For in vivo experiments, expression of vTK was targeted to the livers of FVB/N and SCID/uPA mice. Hepatic sensitivity to GCV was first established in FVB/N mice since these mice do not undergo liver failure inherent to SCID/uPA mice. Hepatic vTK expression was found to be an integral component of GCV-induced pathologic and biochemical alterations and caused death due to liver dysfunction in vTK transgenic FVB/N and non-transplanted SCID/uPA mice. In SCID/uPA mice with humanized liver, vTK/GCV caused death despite extensive replacement of the mouse liver parenchyma with HH (ranging from 32–87%). Surprisingly, vTK/GCV-dependent apoptosis and mitochondrial aberrations were also localized to bystander vTK-negative HH.

**Conclusions/Significance:**

Extensive replacement of mouse liver parenchyma by HH does not provide a secure therapeutic advantage against vTK/GCV-induced cytotoxicity targeted to residual mouse hepatocytes. Functional support by engrafted HH may be secured by strategies aimed at limiting this bystander effect.

## Introduction

The human liver is a crucial organ for pharmacological studies aimed at developing new human medicines. In the preclinical stage, the pharmacokinetics of a drug candidate is investigated using human-derived sources or experimental animals. Because of species differences, human liver microsomes and human hepatocytes (HH) in primary culture are recognized as better tools and are frequently used during drug development. Human liver microsomes can be stored for a few years without the loss of enzyme activities[1], [2], [3] but cannot be used to evaluate the induction potencies of a drug. Induction is often clinically significant and represents the net increase in the levels of one or more drug metabolizing enzymes, as a result of increased *de novo* protein synthesis or protein stabilization[4], [5]. Human hepatocytes express all the drug metabolizing enzymes, but enzyme activities are often reduced by cell culture methods[6], [7]. An artificial human liver is one of the best approaches for predicting human pharmacokinetics and safety in the preclinical stage.

We have developed the SCID/uPA mouse model of human liver, based on transgenic mice in which uPA expression is targeted to hepatocytes (Alb-uPA), and this achievement has advanced our understanding of the in vivo replication properties of Hepatitis C virus[8]. Alb-uPA mice develop hepatocellular disease[9] and likewise, the liver from young SCID/uPA mouse liver initially appears pale. Red foci become visible at approximately 2 weeks old and gradually expand until the pale liver (PL) is replaced by confluent red, regenerative nodules (RN); a process that typically takes 3 months in mice that carry one copy of the uPA transgene[9], [10]. RN have been shown to arise from the proliferation of individual mouse hepatocytes (MH) that have deleted the uPA transgene[9]; an event that is more likely to occur in heterozygous uPA transgenic mice since individual hepatocyts have only one copy of the transgene. However, despite early transplantation[8], HH transplanted into SCID/uPA mice (homozygous for the uPA transgene) will compete with uPA-deficient MH for replacement of the mouse liver parenchyma. As such, variable levels of human chimerism is often achieved[8], [10], [11] and complete humanization of mouse liver has never been achieved. SCID/uPA mice with high levels of human liver chimerism have been shown to retain normal pharmacological responses and therefore are potentially useful for human drug metabolism studies[11]. Given the marked differences in drug metabolism between humans and mice[12], there is a need for complete humanization of SCID/uPA mouse liver.

It is well known that differentiated hepatocytes of adult liver will transiently enter the cell cycle and proliferate to restore lost liver mass after parenchymal damage or resection[13], [14], [15], [16]; a phenomenon that is especially evident following 70% partial hepatectomy (PH)[17]. In Alb-uPA mice, RN have been shown to preferentially respond further to the strong mitotic stimulus of hepatectomy relative to PL[9]. We hypothesized that conditional ablation of residual MH in chimeric liver of SCID/uPA mice would leave only HH capable of this mitotic response and their selective proliferation would promote complete humanization of mouse liver.

The Herpes Simplex Virus type 1 thymidine kinase (vTK) coding region is a component of a strategy for cell-type specific ablation in transgenic animals in response to the pro-drug ganciclovir (GCV) (reviewed in[18]). We reproduced vTK/GCV-induced hepatic failure of mouse liver[19], [20] in SCID/uPA mice for the conditional ablation of residual MH with the intention of producing mice with consistently greater levels of human liver chimerism.

## Results

### Immunohistology of Chimeric Mouse Liver

**Figure 1:**
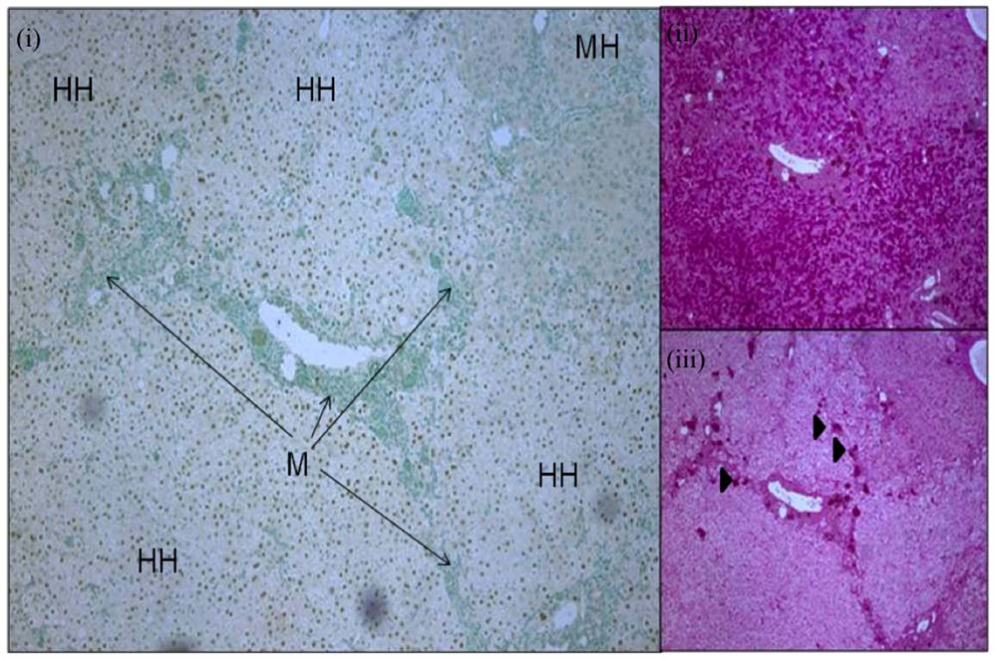
Histology of chimeric SCID/uPA liver at 12 weeks old. Engrafted human hepatocytes (HH, brown nuclei) in paraffin embedded liver sections were distinguished from mouse hepatocytes (MH, blue nuclei) and other murine constituents (M, blue nuclei) by hybridization with fluoresceinated Alu probe (i, left). Serial sections were analyzed by PAS staining (right) without (ii) or with (iii) prior diastase treatment. Ceroid macrophages (iii, arrowheads). (Original magnification ×50).

Histopathological analysis of chimeric liver from a 12 week old SCID/uPA mouse (transplanted at 5 days old),demonstrated two types of lobules comprised of HH or MH (Fig. 1, i) which are separated by murine (M) thin connective tissue and portal tracts (short arrow), where ceroid-laden macrophages cluster (iii, arrowheads). HH are larger than MH and have a pale cytoplasm corresponding to glycogen storage (ii positive by periodic acid Schiff (PAS), and iii negative by diastase PAS,).

### vTK/GCV Cytotoxicity in Huh7 Cells

**Figure 2:**
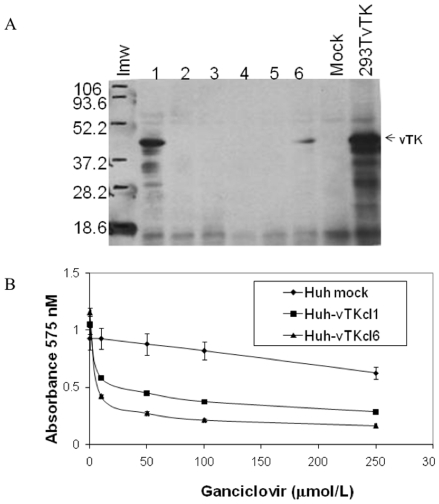
Functional expression of vTK in Huh7 cells. **A.** Huh7 cells were transfected with pCI-vTK and G418-resistant Huh cellular clones (cl) (1–6) were evaluated for vTK expression (∼43 KDa, arrowheads) by immunoblot analysis. Lmw, low molecular weight markers; 293TvTK, 293T cells transiently transfected with pCI-vTK. **B.** In vitro cytotoxicity of GCV in vTK expressing cells. HuhvTKcl1 and HuhvTKcl6 cells were incubated with various concentrations of GCV for 72 hours, followed by cell survival quantitation by MTT assay. Data represent the Mean +/− S.D from quadruplicate cell cultures for each GCV dose. Mock, Huh cells that underwent stable selection after transfection with empty pCIneo vector.

Immunoblot analysis demonstrated Huh7 cellular clones (1 and 6) stably transfected with pCI-vTK expressed vTK protein (Fig. 2A). These vTK expressing cellular clones started to detach 48 h post exposure to a single dose (50 mmolL^−1^) GCV whereas the mock transfected cells grew and proliferated normally. Increased cell death was also assessed by MTT assay (Fig. 2B). Stable expression of vTK in Huh7 cells significantly reduced their survival rate in the presence of the toxic pro-drug GCV.

### vTK/GCV Induced Liver Failure in FVB/N Mice

Female vTK+FVB/N mice (derived from a single female founder) were crossed with wild-type FVB/N males and the vTK status of offspring was determined by vTK PCR-based genotyping of tail biopsies in order to identify vTK+ and vTK-FVB/N mice from which an experimental cohort to test vTK/GCV-inducible hepatic injury was selected (Table 1). Experimental mice had to be sacrificed after 5 days of dosing due to GCV-induced health crisis seen only with vTK+ mice. Baseline serum aminotransferases ranged from 45–55 and 73–106 IU/mL for ALT and AST, respectively and were found to be substantially increased (to >200 and >800 IU/mL, for ALT and AST, respectively) in vTK+ mice that received 25 and 50 mg/kg GCV and in whom GCV dosing had the most severe impact (Table 1). Creatinine concentrations remained within normal ranges (30–58 mmol/L) indicating normal kidney function. The appearance of livers from vTK+ FVB/N mice ranged from very red (as seen with mice that remained healthy, +++) to very pale (as seen with the most severlely impacted, +). The health and gross appearance of livers from vTK-FVB/N mice remained normal at all GCV doses.

**Table 1:** vTK/GCV induced liver failure in FVB/N mice.

Animal ID	Gender (M/F)	vTK Status	GCV Dose (mg/kg)	Health Status	Liver Color	ALT (IU/L) T_0_ T_f_		AST (IU/L) T_0_ T_f_	
3.2	M	−	0	+++	Red	53	49	88	94
3.18	M	−	10	+++	Red	55	51	75	84
3.19	M	−	10	+++	Red	54	52	99	79
3.12	F	−	25	+++	Red	48	44	100	88
3.14	F	−	25	+++	Red	45	49	102	101
3.1	F	−	50	+++	Red	51	48	79	87
3.3	M	+	0	+++	Red	48	52	81	101
3.21	M	+	10	++	Pale Red	51	53	91	83
3.11	F	+	25	+	Pale	53	230	88	896
3.13	M	+	25	+	Pale	49	247	106	1036
3.9	M	+	50	+	Very Pale	48	304	73	1211
3.10	F	+	50	+	Very Pale	51	309	89	1214

GCV was administered to vTK+ and vTK-FVB/N mice for 5 days. Health status ranged from excellent (+++), to poor (++), to critical. ALT and AST levels were determined for blood draws obtained at 1 hour prior to (T_0_) and at the end of (T_f_) the dosing period. Livers ranged from healthy/normal (red), to very pale at the end of the study.

### Tissue Distribution of vTK in vTK+FVB/N Mice

**Figure 3:**
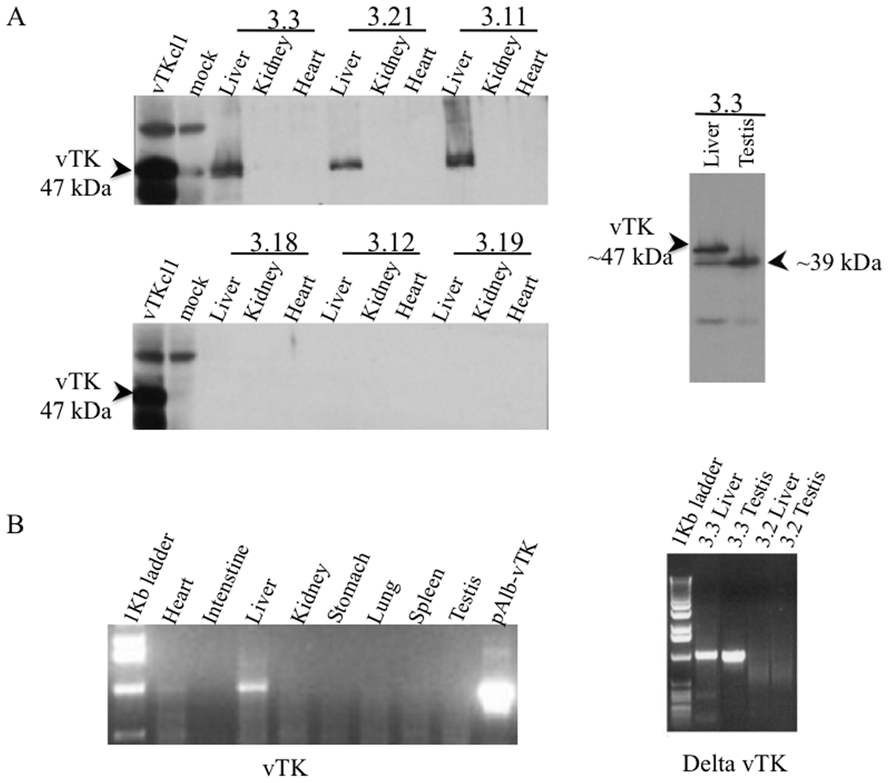
Tissue distribution of vTK in transgenic mice. **A.** Expression of vTK protein (47 kDa) in various tissues from experimental FVB/N mice (listed in Table 1) were examined by immunoblot analysis (as shown for vTK+ mice 3.3, 3.21 and 3.11 and vTK− mice 3.18, 3.12, and 3.12, left panel). A smaller vTK immunoreactive protein (∼39 kDa) was identified in the testis of vTK+ mice. (right panel). **B.** Expression of vTK mRNA was evaluated in several tissues obtained from vTK+ mice (as shown for 3.3, left). Expression of delta-vTK mRNA was evaluated in the liver and testis of vTK+ (as shown fo 3.3, right) and vTK− (as shown fo 3.2, right) mice.

Various tissues from vTK+FVB/N mice underwent immunoblot and RT-PCR analysis for vTK expression (Fig. 3). Full-length vTK protein (47 kDa) was detected exclusively in the livers of vTK+ mice and was absent from vTK- mice (Fig. 3A, left). A smaller immunoreactive protein was detected in the testis of vTK+ mice (Fig. 3A, right) but was absent from testis from vTK- mice (not shown). RT-PCR for vTK (Fig. 3B, left) and delta-vTK (Fig. 3B, right) confirmed the presence of full length vTK mRNA in the liver and delta-vTK mRNA in the testis from vTK+ mice (as shown for mouse vTK+ mouse 3.3). The detection of a similar amplicon in heart was found to be due to genomic DNA contamination of the total RNA prepared from heart tissue since this amplicon disappeared when the total RNA was treated with DNase (not shown). An amplicon corresponding to delta-vTK was also detected in the liver of vTK+ mice and was expected since the primers for delta-vTK RT-PCR are expected to amplify cDNA generated from full length vTK transcripts (refer to Materials and Methods section). Transcripts for delta-vTK were absent in the testis and liver from vTK- mice (B, right for vTK- mouse 3.2).

### vTK/GCV Pathological Changes in FVB/N Mouse Liver

**Figure 4:**
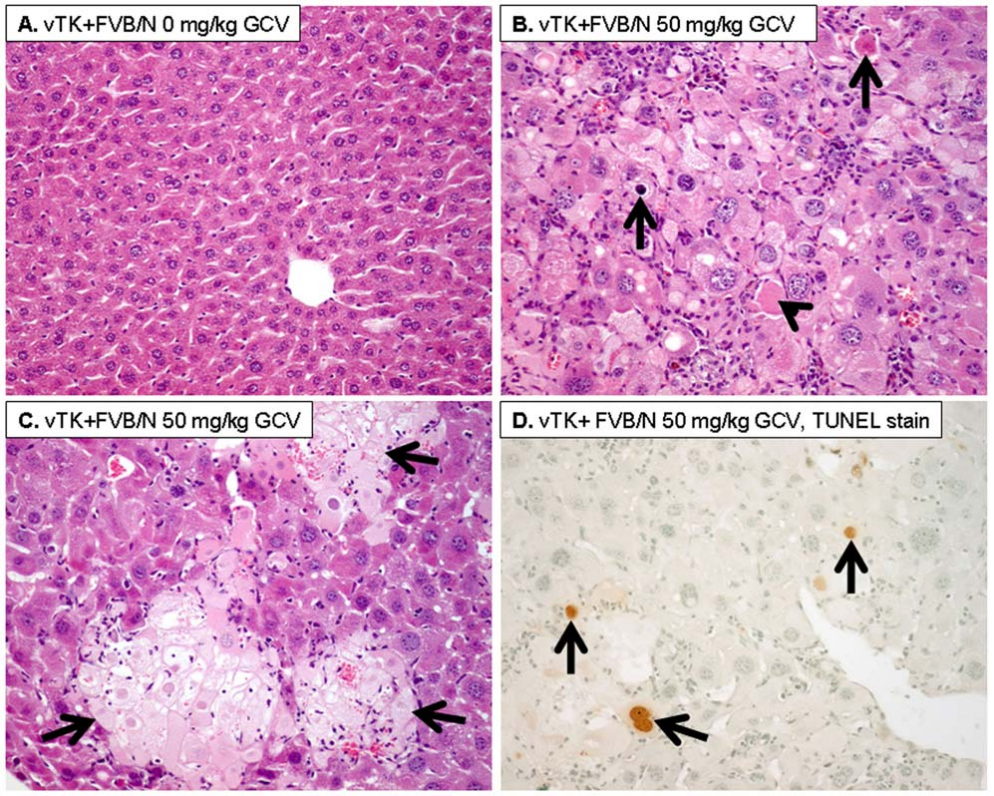
GCV induced histopathological changes. **A.** vTK+FVB/N mice that did not receive GCV showed normal liver histology. **B–C.** vTK+FVB/N mice that received 50 mg/kg GCV showed increased cytoplasmic and nuclear enlargement with increased acidophilic bodies (B, arrowhead), apoptotic bodies (B, arrow), and areas of confluent necrosis (C, arrows) (A–C:Hematoxylin and eosin, original magnification ×200). **D.** TUNEL immunostaining showing increased apoptotic nuclei (arrows) in livers post GCV (Immunoperoxidase, original magnification ×200).

vTK+ FVB/N livers with no GCV treatment showed normal histology (Fig. 4A). However, GCV-treated vTK+FVB/N mouse livers showed diffuse cytoplasmic and nuclear enlargement in hepatocytes with patchy areas of confluent necrosis and abundant acidophilic bodies and apoptotic bodies (Fig. 4B–C). In addition, a prominent mononuclear inflammatory cell infiltration in lobular and portal areas was observed. TUNEL stain showed increased apoptotic nuclei in livers from mice that received 25 and 50 mg/kg GCV (Fig. 4D) and in whom GCV had the most severe impact (Table 1, +) in comparison to vTK+FVB/N with no GCV treatment (not shown) (10% versus 0% positive nuclei, respectively).

### Impact of vTK/GCV in SCID/uPA Mice

Initially, GCV (0, 25, 50 and 100 mg/kg) was administered to non-transplanted vTK+ and vTK− SCID/uPA mice. Dosing was terminated after 10 days due to health crisis seen with vTK+ mice that received 50 and 100 mg/kg GCV doses; only these mice had substantially elevated serum aminotransferases. For vTK− mice (n = 4), the baseline serum ALT and AST levels were 96 +/− 12 IU/L and 293 +/− 23 IU/L, respectively. After the dosing period, only vTK+ mice that received 50 and 100 mg/kg GCV doses had substantially elevated ALT/AST levels increasing to 205/801 IU/L and 238/1003 IU/L, respectively. Creatinine levels remained in the normal range for all mice (not shown).

**Figure 5:**
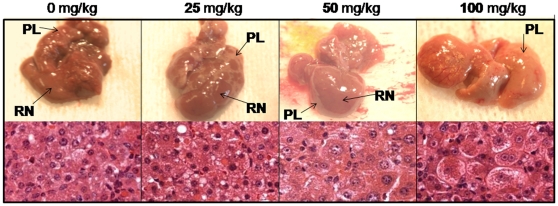
Impact of vTK/GCV on non transplanted SCID/uPA mouse liver. Gross Appearance (upper panels) and H&E staining (lower panels) of livers from non-transplanted and age-matched vTK+SCID/uPA mice (3 months old) treated with 0, 25, 50 and 100 mg/kg GCV (i.p. every 48 h for 10 days). RN, regenerative nodules; PL, pale liver.

**Figure 6:**
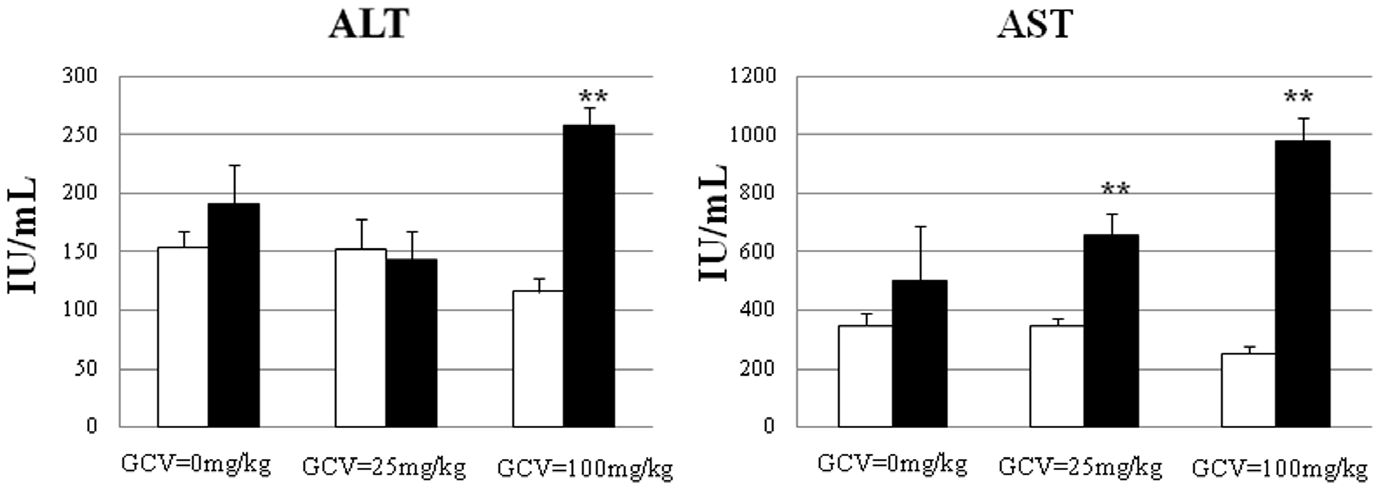
Impact of vTK/GCV on serum aminotranserases levels in chimeric SCID/uPA mice. ALT (left) and AST (right) concentrations in blood sampled from experimental mice (Table 2) 1 h prior to (baseline, white bars) and at the end (endpoint, black bars) of the GCV treatment period (after 14 days of GCV administration with the exception of V241 whose endpoint sampling was after 10 days of GCV administration at which time V241 exhibited health crisis and had to be euthanized). Data represent Mean +/− S.E.M. P<.05 was considered to be significantly different endpoint levels relative to baseline (**).

Like (vTK−)SCID/uPA mouse liver, the majority of the liver parenchyma in vTK+SCID/uPA mice was occupied by intensely red colored RN surrounded by pale liver parenchyma (Fig. 5, upper panels). RN appeared to be particularly sensitive to GCV since their redness could be diminished by GCV treatment; the livers of vTK+ mice that received the highest dose of GCV were universally pale and the discrimination between RN and surrounding pale liver parenchyma was less evident. Like their FVB/N counterparts, the livers from vTK+ SCID/uPA mice that received GCV treatment displayed hepatocytes with dffuse cytoplasmic and nuclear enlargemenet with mononuclear inflammatory cell infiltration (Fig. 5, lower panels). Areas of necrosis, acidophilic bodies, necrosis and apoptotic bodies were also featured in these livers but were absent from vTK− SCID/uPA mice and vTK+ SCID/uPA mice that did not receive GCV (not shown). Having confirmed GCV sensitivity in non-transplanted mice, GCV was administered to transplanted vTK+ and vTK-SCID/uPA mice that had established levels of human chimerism (summarized in Table 2). The average pre-dose ALT/AST values for all vTK− and vTK+ chimeric mice were within the range of the non-transplanted vTK-SCID/uPA mice. These were 135 +/− 40 IU/L and 313 +/− 72 IU/L, respectively. The experiment had to be terminated after 14 days of GCV administration due to health crisis seen only in vTK+ chimeric mice (with the exception of mouse v241 which had to be euthanized after 10 days of GCV administration). These mice had significantly elevated aminotransferases relative to experimental vTK− chimeric mice (Fig. 6). Serum creatinine concentrations remained in the normal range for all chimeric experimental mice (not shown).

**Table 2:** vTK/GCV induced liver failure in chimeric SCID/uPA mice.

Animal ID	Gender (M/F)	vTK Status	GCV Dose (mg/kg)	Baseline hAAT (µg/ml)	Health Status	Pre-treatment Body Weight (g)	Post-treatment Body Weight (g)
V241	M	+	100	129	+	15.3	ND
V243	M	+	100	684	+	12.25	8.65
V233	M	+	100	13	+	15.85	11.20
V232	M	+	25	130	+	15.70	10.40
V236	M	+	25	207	+	18.10	12.75
V250	F	+	25	37	+	10.15	7.70
V237	M	+	25	72	+	10.50	10.90
V234	F	+	0	270	+++	14.50	13.50
V239	F	+	0	14	+++	16.65	16.85
V240	F	+	0	35	+++	13.30	12.85
V251	F	+	0	281	+++	8.90	9.35
V252	F	+	0	19	+++	10.85	11.30
V235	F	−	100	53	+++	14.70	13.45
V238	F	−	100	317	+++	14.80	13.85
V244	F	−	100	133	+++	11.70	9.45
V245	F	−	100	117	+++	11.60	9.15

GCV dosing in chimeric SCID/uPA/vTK and vTK− littermates. The degree of human chimerism was determined by serum ELISA for hAAT of blood drawn at 8 weeks post-transplant (baseline), just 1 hour prior to administration of 0, 25 and 100 mg/kg GCV (i.p.) every 48 hours for up to 14 days at which time the experiment had to be terminated, and all animals sacrificed, due to morbidity of vTK expressing mice that received 25 and 100 mg/kg GCV (with the exception of V241, which had to be euthanized after 10 days of GCV dosing). Health status at the end of the treatment period ranged from excellent (+++), to poor (++), to critical and requiring euthenasia (+) as displayed by weight loss (>10% of pretreatment body mass), decreased activity and lethargy, ruffled fur, and hunched posture. ND: not determined.

**Figure 7:**
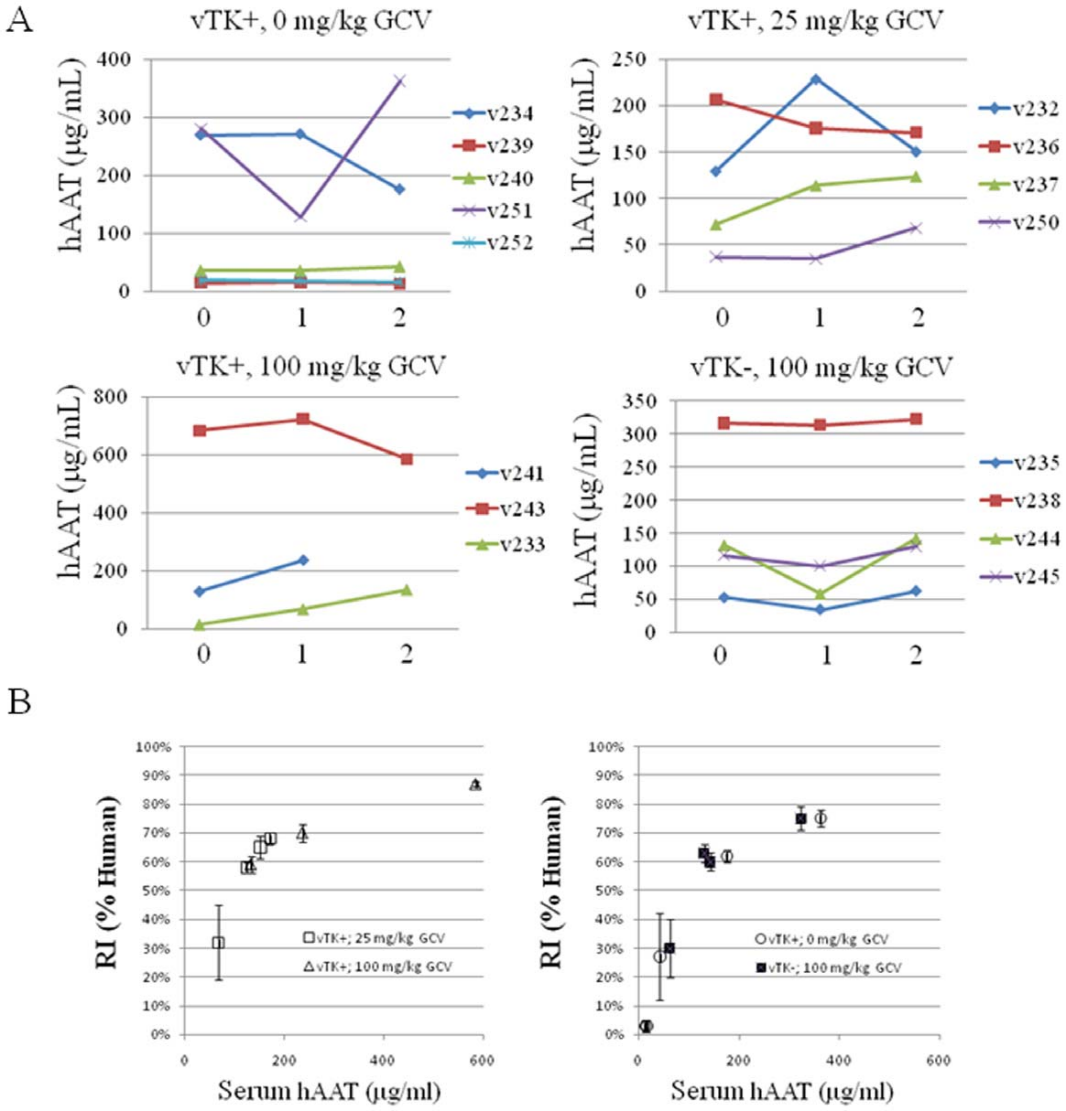
Impact of vTK/GCV on hAAT production and human hepatocyte engraftement in chimeric SCID/uPA mice. **A.** Baseline blood samples were obtained from experimental mice (Table 2) 1 h prior to the commencement of GCV dosing (t_0_) for hAAT analysis. Subsequent samples were obtained from all chimeric mice for hAAT analysis once weekly for 2 weeks with the exception of v241 which had to be euthanized after 10 days of GCV dosing. **B.** A correlation plot of final hAAT concentration vs. replacement index (RI) was constructed for those experimental mice (Table 2) that were severely impacted by GCV (left) and those that remained healthy during the course of dosing period (right). RI was determined as the ratio of the area occupied by Alu-positive HH relative to the entire area examined in the *in situ* hybridization sections expressed as percent. Data represent the mean RI +/− SEM of at least 3 separate lobes).

Serum hAAT levels for the majority of transplanted animals (13 of 16 mice) were higher at the end of the GCV treatment period relative to baseline (Fig. 7A). There was a good correlation between hAAT levels and replacement index (RI) for histologically integrated HH (Fig. 7B) and this did not appear to be impacted by health status since correlations were similar between chimeric mice that remained healthy and thrived during the course of GCV dosing (left) and those that were severely impacted and required euthanasia (right). These results suggest continued expansion of HH in chimeric mice during the course of GCV administration but do not exclude the involvement of GCV in a more subtle effect on engrafted HH. Therefore, increased serum levels of hAAT, ALT, and AST may reflect bystander killing of vTK-negative HH.

### Impact of vTK/GCV on Gross Appearance, Histopathology and Ultrastructural Architecture of Chimeric Liver

**Figure 8:**
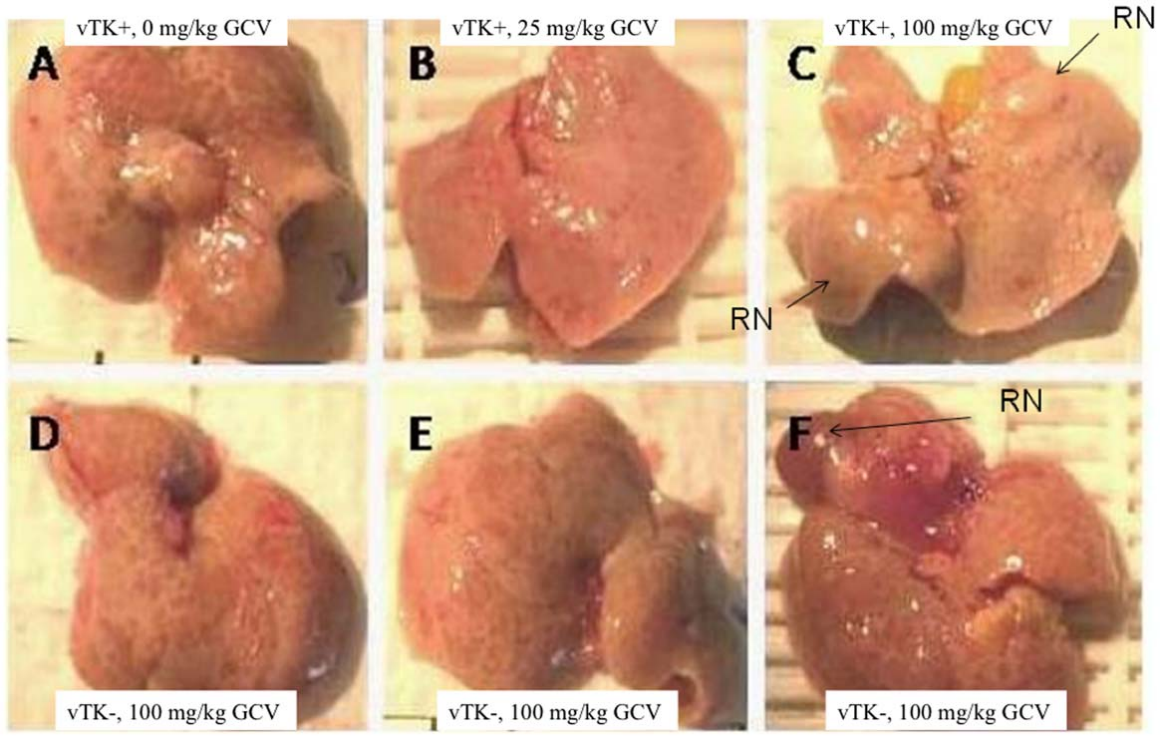
Impact of vTK/GCV on the gross appearance of chimeric SCID/uPA liver. Chimeric livers from vTK− SCID/uPA mice (Table 2) exhibited multiple small red-colored foci (D, E, and F for mice V245, V244 and V238, respectively) that were clearly evident in liver from chimeric vTK+ SCID/uPA mice that did not receive GCV (A for mouse V250), but were diminished in chimeric livers from vTK+ SCID/uPA mice that received 25 and 100 mg/kg GCV doses (B and C, respectively). Occasionally, chimeric livers from chimeric mice also exhibited large 3-dimensional regenerative nodules (RN).

Chimeric livers from vTK+ mice that did not receive GCV (Fig. 8A) had similar gross appearance and architecture as chimeric livers sampled from vTK− mice that received 100mg/kg GCV (Fig. 8D–F,). These livers are characterized by multiple red colored foci. Occasionally larger red regenerative nodules (RN) typically seen in livers of age-matched non-transplanted mice (as shown in Fig. 5) were also seen (Fig. 8F). The combination of vTK/GCV resulted in chimeric livers with a more pale appearance with a substantial reduction in the numbers of red foci that could be discriminated from the pale liver parenchymal background (Fig. 8B and C for vTK+ chimeric mice given 25 and 100 mg/kg GCV doses, respectively). Even large 3-dimentional RN appeared remarkably pale (Fig. 8C) when compared with the typically red RN seen in chimeric SCID/uPA mice (as seen with chimeric vTK− SCID/uPA liver, Fig. 8F).

**Figure 9:**
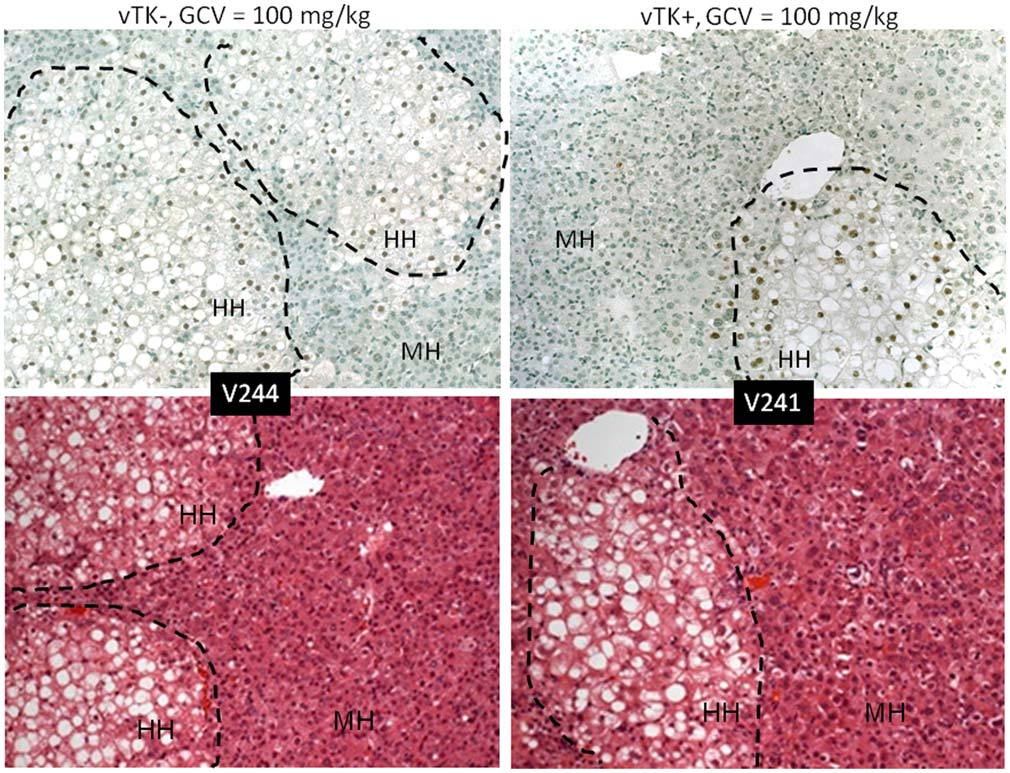
Histopathological changes induced by vTK/GCV in chimeric SCID/uPA mice. Engrafted human hepatocytes (HH, bordered by dashed line) in paraffin embedded liver sections from experimental mice (Table 2) were identified by hybridization with fluoresceinated Alu probe (top). Serial sections were analyzed by H& E staining (bottom). MH; mouse hepatoctyes. Original magnification ×100.

**Figure 10:**
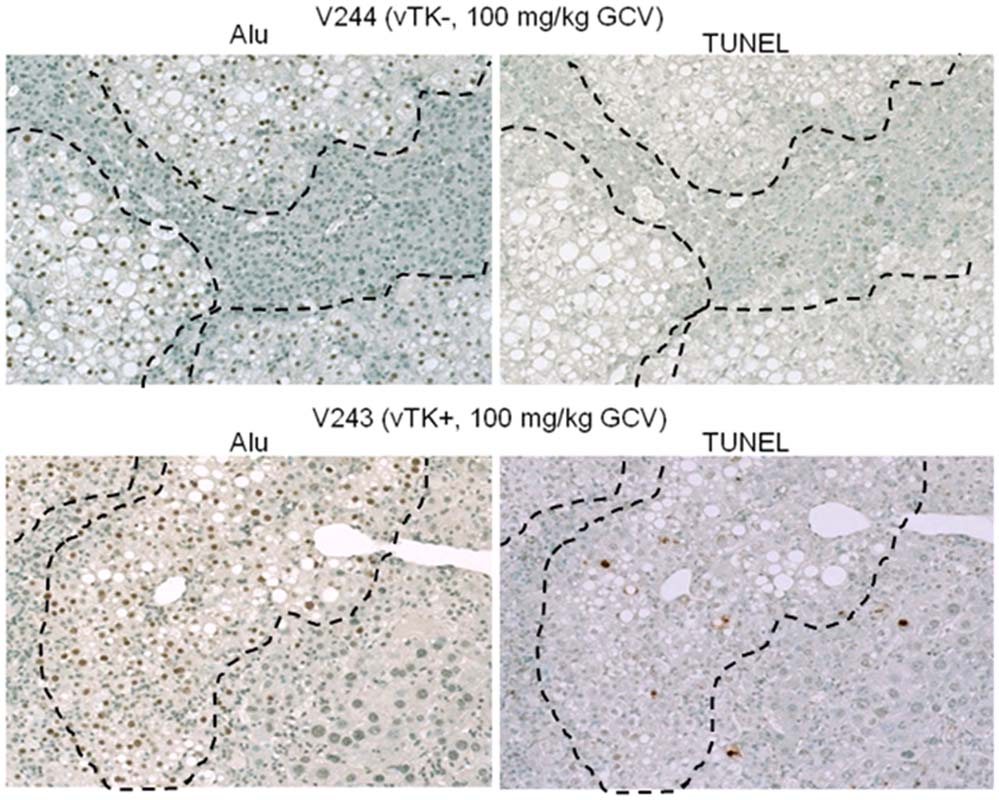
Induction of apoptosis by vTK/GCV in chimeric SCID/uPA mice. Engrafted human hepatocytes (bordered by dashed line) in paraffin embedded liver sections from experimental mice (Table 2) were identified by hybridization with fluoresceinated Alu probe (left). Indirect TUNEL analysis of DNA fragmentation was performed on serial sections (right). Original magnification ×100.

Diffuse cytoplasmic and nuclear enlargement were readily detected in the MH populations of chimeric livers from vTK+ SCID/uPA mice that received GCV but had normal histology in chimeric livers from vTK− SCID/uPA (despite 100 mg/kg GCV dosing, Fig. 9). Increased apoptotic and acidophilic bodies were detected in both MH and HH populations of chimeric livers from vTK+ SCID/uPA that received GCV compared to those from mice that did not receive GCV and vTK− SCID/uPA mice (not shown). Serial Alu and TUNEL staining revealed TUNEL-positive apoptotic nuclei in both MH and HH populations of chimeric liver from vTK+ mice that received GCV (Fig. 10). By contrast, TUNEL-positive cells were rarely seen in chimeric livers from vTK+ SCID/uPA mice that did not receive GCV and vTK− chimeric mice (15% versus 1%, respectively, not shown).

**Figure 11:**
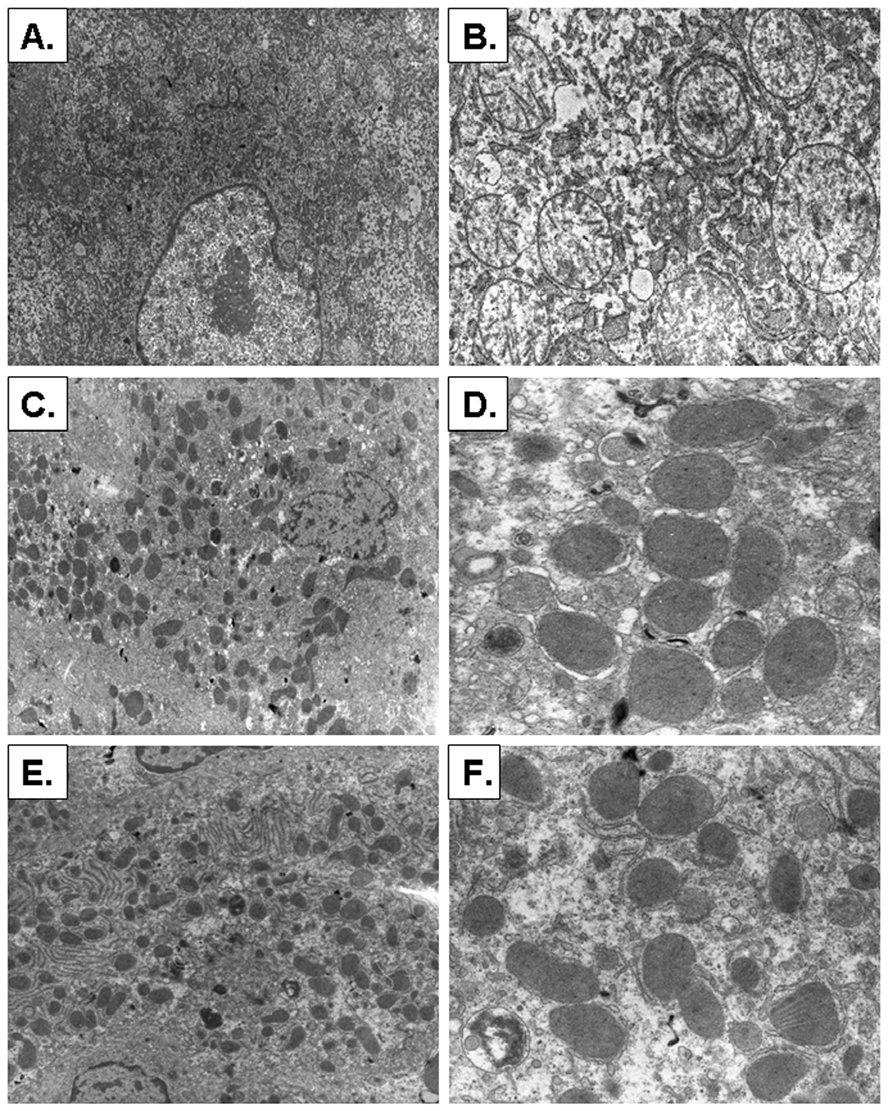
Ultrastructural changes after GCV treatment. **A–B.** Chimeric vTK+ SCID/uPA livers after GCV treatment (100 mg/kg) showing swollen electron lucent mitochondria (A) with aberrant cristae and matrices (B). **C–D.** vTK+ SCID/uPA livers with no GCV showing normal cellular (C) and mitochondrial (D) ultrastructure. **E–F.** vTK− SCID/uPA livers showing normal cellular (E) and mitochondrial (F) ultrastructure (Uranyl acetate-lead citrate, original magnification, A,C,E: ×1500; B,D,F: ×3600). These micrographs represent ultrastructural features that are present in 10 biopsies with a minimum of 5 random fields examined per mouse.

Ultrastructural analysis was performed by TEM (Fig. 11). GCV induced swollen electron lucent mitochondria with aberrant cristae and matrices (Fig. 11A,B). We did not observe other ultrastructural changes (Figure 11A). In contrast, livers from vTK+ SCID/uPA mice with no GCV (Fig. 11C,D) or vTK− SCID/uPA mice (Fig. 11E,F) had normal ultrastructural morphology with unremarkable mitochondrial matrix and cristae. Ultrastructural analysis performed with biopsies obtained from experimental chimeric vTK+ and vTK− SCID/uPA mice (having RI>60%) demonstrate aberrant mitochondrial features in hepatocytes from chimeric vTK+ SCID/uPA mice that were administered GCV (not shown).

## Discussion

The vTK/GCV strategy for cell-specific ablation in transgenic animals is well documented[18]. This study demonstrates the efficacy of this strategy in humanized SCID/uPA mouse liver for the selective ablation of parenchymal MH to promote complete humanization of SCID/uPA mouse liver. Hepatic sensitivity to GCV was first established in vTK+FVB/N mice since the development of hepatocellular disease, inherent to SCID/uPA mice[8], [9], was not a confounding factor. Hepatic vTK expression was found to be an integral component of GCV-induced pathologic and biochemical alterations and caused death due to liver dysfunction, similar to other vTK/GCV mouse models of liver failure[19], [20] and human liver disease caused by acute to subacute toxin-mediated irreversible hepatocyte damage[21]. The apparent sterility of male vTK+FVB/N mice has been described previously for other vTK-transgenic mice and is likely due to the expression of delta vTK in the testes shown to arise from an alternate transcriptional start site[22], [23], [24], [25]. Inheritance of the vTK minigene by SCID/uPA mice did not appear to impact inherent ability of endogenous MH to develop into RN since by 3 months old, vTK+SCID/uPA mouse liver had architectural features that typically mark the hepatocellular disease seen with age-matched SCID/uPA mice[9], [26]. Like their vTK+FVB/N ancestors, biochemical and pathologic changes in the liver could be regulated by GCV dose, and caused death due to liver dysfunction. Most notable were the gross and pathologic changes that occurred with RN. It is presumed that proliferating vTK expressing cells are particularly more sensitive to GCV than quiescent cells[18] since cells expressing vTK convert the nontoxic prodrug GCV into GCV-triphosphate, which in turn leads to chain termination and single-strand breakage upon incorporation into DNA and ultimately cell death due to apoptosis[27]. GCV sensitivity of RN is not surprising since they have been shown to arise from the clonal expansion of MH that have an enhanced proliferative capacity due to deletion of the uPA transgene[9]. The vTK/GCV combination also killed normally non-proliferating liver parenchymal cells of adult FVB/N mice and this observation has been corroborated by several reports from experimental animal models and from clinical trials suggesting that proliferation is not a requisite for vTK/GCV-induced toxicity[28], [29], [30], [31], [32]. Therefore, it is likely that diseased PL surrounding RN in vTK+SCID/uPA mouse liver also underwent further degeneration from the cytotoxic effects of GCV.

Established human chimerism had only a minor therapeutic advantage in the setting of GCV induced mouse liver failure; possibly due to HH survival and continued expansion. However, despite replacement of up to 87% of the endogenous parenchyma with HH, GCV still induced liver failure and caused death. The known safe limit for experimental liver resection in mice is 70% PH, versus 90% PH, which was found to be lethal in all mice due to severe acute liver failure[17]. This raises the question about whether a humanized liver could replace all necessary mouse liver functions. Limited survival beyond 3 months post-transplant has been described for chimeric SCID/uPA mice with RI>50%[11] but this was due to severe insult to host tissues induced by HH-secreted complement factor[11]. Survival of chimeric mice with RI>50% was achieved by administering a drug that has anti-human complement factor activity; the yield of healthy chimeric mice with RI>70% was 32% with the maximum RI as high as 96%[11]. Although these authors also achieved variable levels of human chimerism, the survival of healthy mice with up to 96% human chimerism strongly suggests that the capacity to satisfy metabolic demand is indeed securable by engrafted HH. In this study, we demonstrate that the capacity of engrafted vTK-negative HH to satisfy metabolic demands is compromised by vTK/GCV induced damage within residual murine liver, even in mice with up to 87% human chimerism. Our findings have been corroborated by at least one other study in which wild-type MH transplanted into Alb-vTK immune competent mice continued to expand during GCV administration, but a consistent therapeutic advantage in rescuing mice from GCV-induced liver disease could not be demonstrated[19]. These observations are interesting with respect to the known ‘bystander effect’ (BE) of vTK-expressing cells incubated with GCV to induce apoptosis in neighboring vTK-negative cells[33], [34], [35], [36], [37], [38], [39]. Generally speaking, the BE is due to intercellular transfer of the activated pro-drug via gap junctions thought to be restricted to dividing cells[38], [40] and has been shown to occur *in vivo* during the treatment of gliomas, sarcomas, or carcinomas[41], [42] and in clinical trials on the assessment of vTK/GCV gene therapy for the treatment of brain tumors[43], [44], [45]. Phagocytosis of material from dying HSV-tk-expressing cells has also been suggested as a potential mechanism[40] but no functional evidence has been presented to date[18]. An immunological component to the BE, involving CD4+ and CD8+ lymphocyte infiltration, has been described[46], [47] and thought to absent when experiments are conducted in immune-compromised animals[48] such as SCID/uPA mice[8]. Many of the histopathological features that characterized vTK/GCV induced liver failure in FVB/N mice and non-transplanted SCID/uPA mice were also localized to the HH populations of chimeric liver. The paucity of TUNEL-positive apoptotic MH and HH likely precludes their involvement in vTK/GCV-induced hepatic failure in chimeric mice. Given that hepatocytes harbor up to 2000 mitochondria per cell and the oxidative phosphorylation capacity of animal tissues is regulated primarily through replication and transcription of mitochondrial DNA[49], any perturbation of mitochondrial integrity can lead to cell death[49]. For SCID/uPA mice, normal hepatic capacity is restored by the development of RN, be they from uPA-deficient MH or HH[8], [9]. Ultrastructural analysis revealed that vTK/GCV induced severe mitochondrial aberrations, similar to those reported for liver from rats that received combined administration of Ad.CMVtk and GCV and in whom hepatotoxicity and mortality correlated with the accumulation of phosphorylated GCV in the mitochondria, a drop in mitochondrial membrane potential and a decrease in mitochondrial DNA[50]. Apoptosis and aberrant mitochondria in bystander HH, as far as we know, is the first indication that functional metabolic coorperation between MH and HH exists in chimeric SCID/uPA mouse liver. Strategies aimed at prevention of the BE will likely facilitate the use of a vTK/GCV system for the development of a mouse model with a completely humanized liver.

## Materials and Methods

### Ethics Statement: Animals

All mice were housed in a virus-free/antigen-free environment, and cared for in accordance with Canadian Council on Animal Care (1993) guidelines. Experimental protocols were reviewed and approved by the University of Alberta Health Sciences Animal Welfare Committee.

### Cell Lines

Huh7 and 293T cells (American Type Culture Collection, Rockville, MD) were maintained in Dulbecco's Modified Eagle's Medium containing 10% heat-inactivated fetal calf serum, 100 Units of penicillin G sodium per ml, and 100 µg of streptomycin sulfate per ml (DMEM-10).

### Plasmids

The HSV*tk* coding region (vTK) was obtained by PCR amplification from a plasmid containing the entire HSV-1 genome (pTK173, a gift from Dr. Jim Smiley, University of Alberta) with vTK/F (5′-GCGCTCTAG*A* *TGGCTTCGTACCCCTGC*-3′) derived from nt 1–18 (Italicized, beginning with the ATG start codon) and vTK/R (5′-CGCGTCTAGA *TC AGTTAGCCTCCCCCAT*-3′) derived from the antisense strand for nt 1111–1131 (Italicized) of the full-length vTK open reading frame (GenBank: AF303108.1, 1131 bp). PCR reactions were performed in 25-ul reaction mixtures using the following conditions: 90 s at 95°C, then 28 cycles of 45 s at 95°C, 45 s at 58°C and 60 s at 72°C, and finally 3 min at 72 C. Following digestion with Xba I, the vTK amplicon (1131 bp) subcloned into the Xba I sites of pCIneo (Promega), to generate pCI-vTK for expression of vTK in mammalian cells, and pAlbHGF in place of HGF and downstream from a murine albumin promoter and enhancer sequence (Alb p/e)[51] to generate pAlb-vTK, for targeted expression of vTK to mouse liver. Sequence and orientation of cloned vTK was confirmed by sequencing (University of Alberta, DNA Sequencing Core Facility).

### vTK/GCV Cytotoxicity in Huh7 Cells

Huh7 and 293T cells were transfected with pCI-vTK using Lipofectamine 2000 (Invitrogen, Burlington, ON, Canada) and G418 (Invitrogen, Burlington, ON, Canada)-resistant cellular clones were selected according to the manufacturers' directions. Cellular expression of vTK protein was detected by immunoblot analysis essentially as described[52] using a rabbit polyclonal anti-vTK antibody (a gift from Dr. Jim Smiley, University of Alberta).

Cytotoxicity of GCV on cell cultures was determined using Cell Proliferation Kit I (MTT) (Roche, Mississauga, ON) according to the manufacturer's instructions.

### vTK Transgenic Mice

pAlb-vTK was digested with BstXI and HindIII to yield a 5134 bp minigene containing the murine Alb p/e with the downstream vTK coding region which was microinjected into fertilized oocytes prior to their transfer into the oviducts of pseudopregnant FVB/N mice using standard procedures[53]. Transgenic (vTK+) and non-transgenic (vTK−) offspring were identified by PCR of genomic DNA extracted from tail tissue (DNeasy, Qiagen) using vTK/F and vTK/R primers and the PCR conditions described above. All attempts to breed with male vTK+ mice were unsuccessful. Female vTK+ FVB/N mice were used to breed with male SCID/uPA (homozygous for the SCID mutation and homozygous for the uPA transgene) mice. vTK expression in the SCID(+,−)/uPA(+/−) (heterozygous for the SCID mutation and hemizygous for the uPA transgene) offspring was confirmed by PCR of tail DNA and the vTK+ female offspring were used for interbreeding with SCID/uPA male mice. Zygosity of SCID mutation and uPA transgene was determined in offspring by established PCR based strategies. For SCID genotyping, the forward primer was oIMR0803 (5′-ggA AAA gAA TTg gTA TCC AC-3′) [54] and the reverse primer was oIMR0804 (5′-AgT TAT AAC AgC Tgg gTT ggC -3′)[55]. PCR was performed in 50 ul reaction volumes using the following conditions: 94°C for 1.5 min, then 35 cycles of 94°C for 30 s, 53°C for 30 s and 72°C 30 sec, and finally 72°C for 2 min. The PCR amplified a 79 bp DNA fragment from both alleles which was subjected to digestion for 2 h at 37 C with AluI. After AluI digestion, products from the wild type allele are predicted to be 68 and 11 bp, and the products from the SCID allele are predicted to be 38, 28, and 11 bp. Digestion products were visualized by ethidium bromide staining following electrophoresis on a 4% agarose gel. Genotyping for the uPA transgene was done using a multiplex PCR procedure as described by Meuleman et al[56]. Only vTK+ and vTK− SCID/uPA (homozygous for both the SCID mutation and the uPA transgene) mice were used for testing vTK/GCV inducible liver failure in chimeric (transplanted) and non-transplanted mice.

### Induction of Liver Damage

A range of GCV dosage concentrations (ranging from 0 to 100 mg GCV/kg body weight) was used to produce groups of mice with variably severe liver disease essentially as described[19]. GCV was administered (i.p) every 48 h to mice at 12 weeks old. All mice were monitored daily and were sacrificed if they showed signs of metabolic crisis typified by weight loss, decreased activity, ruffled fur, and hunched posture.

### Transplantation of Mice

Ethical approval for human tissue was obtained from the University of Alberta, Faculty of Medicine Research Ethics Board, and informed consent was obtained from all human tissue donors. Hepatocytes were collagenase isolated from phosphate-buffered saline flushed normal human liver segments obtained from a patient undergoing liver resection from liver cancer and Percoll purified then injected into the inferior pole of the spleen of 7-day old mice essentially as described[8], [57]. Mice were monitored weekly beginning at 8 weeks post transplant for engraftment success using a serum-based hAAT ELISA[8], [57].

### Biochemical Analysis of Blood

Peripheral blood was sampled from the tail vein just 1 h prior to the commencement of GCV dosing, then once weekly during the course of GCV treatmuent. Serum samples underwent analysis for human alpha-1 antitrypsin (hAAT) using methods we have established previously[8], [57] and for levels of aminotransferases (ALT and AST) and creatinine (performed by the Central Laboratory for Veterinarians, Ltd. Edmonton, AB).

### Tissue Procedures

Tissues were removed, washed with cold PBS, frozen in liquid nitrogen and stored at −80°C until processed. Protein homogenates were prepared essentially as described[58] and analyzed for vTK protein expression by immunoblot analysis (described above). Total RNA was isolated from frozen tissue using Trizol® reagent (Life Technologies, Inc., Burlington, ON, Canada) according to the manufacturer's instructions. First-strand cDNA synthesis from 2 µg of total RNA was performed using SuperScript™ II reverse transcriptase (Invitrogen, Burlington, ON, Canada) primed by vTK/R. PCR was performed using vTK/F and vTK/R primers as described above. For detection of a 5′-truncated vTK RNA (delta vTK), a different forward primer was used in the RT-PCR reaction. The primer used was 5′-tgcc cacgctactg cgg-3′ (delta-vTK/F) corresponding to nt 137–153 of the vTK coding region. Freshly isolated liver samples were fixed in formalin, embedded in paraffin, sectioned, and stained with hematoxylin and eosin (H&E), periodic acid-Schiff's (PAS) with or without diastase. Apoptotic cells were detected *in situ* by the indirect TUNEL method using the ApopTag® Peroxidase In Situ Apoptosis Detection Kit (Chemicon, S7100, Temecula, CA) according to the manufacturer's instructions. Engrafted HH in paraffin embedded liver sections were identified using the Super Sensitive ISH Detection System (Biogenex, San Ramon, CA) with fluoresceinated Alu probe (cat # HK844-2K, Biogenex, San Ramon, CA) according to the manufacturer's instructions.

### Electron Microscopy

Tissues were freshly isolated from vTK+ and vTK-SCID/uPA mouse liver by microdissection (10 random biopsies per mouse liver) and processed for transmission electron microscopy (TEM). Tissue for transmission electron microscopy (TEM) was placed rapidly in 1% glutaraldehyde, fixed for at least 1 h at room temperature, and post-fixed in osmium tetroxide, and then processed into plastic. The ultramicrotome was then used to prepare thin sections, which were collected on a copper grid and stained with lead citrate and uranyl acetate [59]. A minimum of 5 random fields per biopsy was examined by TEM.

### Statistics

Comparisons between variables were tested by Student t test of means, the paired Student t test or Mann–Whitney U test, as appropriate. P<0.05 was considered statistically significant.
